# The influence of magnesium ions on the electrophysiological, analgesic, and BDNF-induced neuromodulation of morphine effects in diabetic rats

**DOI:** 10.3389/fphar.2026.1855220

**Published:** 2026-07-01

**Authors:** Przemysław Kurowski, Kamila Kulik, Agnieszka Kowalczyk, Natalia Wróblewska, Jana Kondrat, Filip Świątkowski, Magdalena Bujalska-Zadrożny

**Affiliations:** 1 Laboratory of Physiology and Pathophysiology, Centre for Preclinical Research, The Medical University of Warsaw, Warsaw, Poland; 2 Department of Pharmacotherapy and Pharmaceutical Care, The Medical University of Warsaw, Warsaw, Poland; 3 Faculty of Pharmacy, The Medical University of Warsaw, Warsaw, Poland

**Keywords:** AMPA, magnesium, morphine, neuropathic pain, NMDA, periaqueductal gray (PAG), streptozotocin-induced diabetes

## Abstract

Neuropathic pain is difficult to treat due to the involvement of multiple signaling pathways, including increased expression of sodium channels, decreased expression of potassium channels, heightened neuronal excitability from the activation of N-methyl-D-aspartate receptors (NMDARs), nerve damage leading to changes in neurotrophic factor levels, and reduced opioid efficacy. As a result, the treatment of neuropathic pain often requires a multifaceted approach. It has been shown that magnesium ions (Mg^2+^), which act as physiological antagonists of NMDARs, can augment opioid analgesia in chronic pain. The aim of this study was to assess the influence of Mg^2+^ on the electrophysiological, analgesic, and brain-derived neurotrophic factor (BDNF)-induced neuromodulation of the morphine profile in diabetic rats. Diabetes in male Wistar rats was induced by a single intramuscular injection of streptozotocin (STZ). The Plantar Test was used to assess the effect of Mg^2+^ and morphine co-treatment on STZ-induced hyperalgesia, expressed as increased warm sensitivity of the experimental rat hind paw. *Ex vivo* whole-cell recordings from ventrolateral periaqueductal gray (vlPAG) neurons were conducted to evaluate the frequency of action potentials evoked by incremental depolarizing current steps (current-clamp, 1-s steps). To assess glutamatergic signaling, neurons were voltage-clamped to measure NBQX-sensitive current at −70 mV and NMDA-evoked currents during depolarization in the presence of NMDA/glycine. Changes in BDNF concentrations in PAG structures were measured using an enzyme-linked immunosorbent assay. The results of our study suggest that although magnesium sulfate shows limited analgesic properties when administered alone, it can significantly enhance opioid efficacy when used as an adjunct. Moreover, the co-administration of morphine with magnesium sulfate resulted in a greater reduction in NMDA-evoked currents in diabetic rats compared to either agent alone. Overall, our findings support the concurrent use of Mg^2+^ and morphine in the treatment of neuropathic pain as a mechanism-informed adjunct strategy.

## 1 Introduction

Neuropathic pain is a consequence of damage to central and/or peripheral nervous system neurons and may occur during diabetes. It is characterized by hyperalgesia or allodynia. Damaged sensory neurons in diabetic neuropathy can generate action potentials in the absence of stimuli (Feldman et al., 2019; Żylicz and Krajnik, 2003). While these peripheral changes increase nociceptor excitability, persistent pain also depends on central sensitization within supraspinal circuits, including the periaqueductal gray (PAG) (Heinricher, et al., 2009). Experimental diabetic neuropathy induced by streptozotocin (STZ) is characterized by a progressive evolution of sensory abnormalities. During the early stages of diabetes, animals typically develop hyperalgesia and allodynia, whereas prolonged disease duration may lead to sensory loss and hypoesthesia associated with degeneration of peripheral nerve fibers.

The maintenance of pain may result from increased expression of sodium channels and decreased expression of potassium channels. A time- and concentration-dependent increase in tetrodotoxin-resistant (TTX-resistant) sodium currents has been observed in response to hyperglycemia (Singh et al., 2013). Additionally, high-voltage-activated Ca^2+^ current amplitudes and the activity of T-type channels (Cav3.2) are elevated in small-diameter neurons in diabetes (Hall et al., 2001; Jagodic et al., 2007). Furthermore, decreased voltage-gated potassium (Kv) currents and reduced mRNA levels of IA subunits (Kv1.4, Kv3.4, Kv4.2, and Kv4.3) have been reported in dorsal root ganglion (DRG) neurons of diabetic rats (Cao et al., 2010). These peripheral mechanisms provide the substrate for hyperexcitability at the input stage. However, central nodes such as the PAG integrate and amplify these signals to maintain chronic pain.

These changes are followed by alterations in the release and synthesis of neurotrophic growth factors, which may be linked to neuroregeneration processes (Campbell and Meyer, 2006). The ventrolateral subdivision of the PAG (vlPAG) is a key hub of the descending pain modulatory system, projecting to the rostral ventromedial medulla and shaping spinal nociceptive transmission (Heinricher, et al., 2009). Increased neuronal excitability observed in individuals with neuropathic pain is caused by the activation of N-methyl-D-aspartate receptors (NMDARs). In streptozotocin (STZ)-induced diabetic rats, NMDAR activation has been associated with phosphorylation of the NR1 receptor subunit and the development of hyperalgesia (Guo et al., 2002; Zhou et al., 2019). Moreover, reduced expression of opioid receptors in the spinothalamic pathway and decreased morphine efficacy have been reported in this model (Chen and Pan, 2002; 2003; Chen et al., 2002).

Current pharmacological management of painful diabetic neuropathy includes antidepressants (e.g., duloxetine and tricyclic antidepressants) and gabapentinoids as first-line therapies (Soliman et al., 2025). However, many patients experience only partial pain relief or discontinue treatment because of adverse effects. Although opioid analgesics are generally reserved for later-line treatment due to concerns regarding tolerance, dependence, and other side effects (Soliman et al., 2025), morphine remains one of the most extensively studied opioids in experimental pain research. Importantly, reduced responsiveness to morphine has been consistently observed in STZ-induced diabetic neuropathy (Chen and Pan, 2002; 2003; Chen et al., 2002), making this model particularly useful for investigating mechanisms underlying impaired opioid analgesia. Within vlPAG, µ-opioid receptors (MORs) are abundant on GABAergic interneurons; their activation engages GIRK-mediated inhibition of interneurons, disinhibiting output neurons and promoting antinociception (Chieng and Christie, 1994; Vaughan et al., 1997). Although opioid-induced analgesia may be achieved with higher doses of opioid agonists in this context, such treatment is often accompanied by side effects, including respiratory depression, opioid-induced hyperalgesia, and the development of tolerance and addiction (Algera et al., 2021).

Activation of the glutamatergic system has a significant impact on the development and maintenance of neuropathy; therefore, NMDAR antagonists remain a rational adjuvant strategy for relieving neuropathic pain (Yan et al., 2013). It has been shown that non-competitive NMDAR antagonists, such as ketamine and MK-801, reduce the development of allodynia and hyperalgesia in this type of pain. Unfortunately, clinical use of broad NMDAR antagonists is limited by side effects—including amnesia, sedation, and mental disturbances—due to their low therapeutic index. Consequently, more selective NMDAR antagonists are being investigated to minimize these adverse effects (Kim et al., 2012; Zhou et al., 2011). Importantly, enhanced NMDAR signaling can also blunt opioid efficacy, suggesting mechanistic interplay between glutamatergic and opioid systems (Price et al., 2000).

The involvement of NMDARs in the development of neuropathic pain may also reduce sensitivity to opioids. These findings suggest a strong synergistic effect between morphine and low doses of NMDAR antagonists (Dickenson, 1997). Pharmacological and biochemical studies have clarified the relationship between NMDARs and MORs. Both receptor types are expressed on the dendrites and somata of PAG neurons. Studies by Rodríguez-Muñoz et al. (2012), Sánchez-Blázquez et al. (2010) indicate that MOR is associated with the NMDAR NR1 subunit in the synaptic membrane. Magnesium ions (Mg^2+^) are physiological, voltage-dependent pore blockers of NMDARs. Numerous clinical trials have demonstrated the analgesic effects of Mg^2+^. Administration of magnesium sulfate significantly reduced the need for pain medications and prevented side effects (Shin et al., 2020). Rondon et al. (2010) found that oral magnesium supplementation prevented enhanced phosphorylation of the NMDAR NR1 subunit in diabetic rats. In our previous studies on magnesium sulfate–treated diabetic neuropathic rats, a significant reduction in MOR C-terminal Ser375 phosphorylation, along with decreases in protein kinase A (PKA) and protein kinase C (PKC) activity, was observed (Kulik et al., 2021). Considering the good tolerance and antagonistic properties of Mg^2+^ ions, their use in neuropathic pain treatment appears justified (Begon et al., 2002).

Decreased levels of nerve growth factor, brain-derived neurotrophic factor (BDNF), and insulin-like growth factor 1 have been observed in diabetic neuropathy (Apfel, 1999; Hellweg and Hartung, 1990; Nitta et al., 2002). Because BDNF promotes axonal and dendritic growth and supports neuronal survival (Xu et al., 2000), its reduction may increase neuronal susceptibility to damage during chronic hyperglycemia. BDNF also appears to have pain-modulating properties, because its infusion into the midbrain produces analgesia (Siuciak et al., 1994). However, pronociceptive effects of BDNF have also been described (Sikandar et al., 2018). Primary afferents express both glutamate and BDNF, and stimulation of sensory fibers during neuropathy triggers their release in the spinal cord. After binding to postsynaptic tropomyosin kinase B (TrkB) receptors, BDNF activates phospholipase C and subsequently PKC, which elevates intracellular Ca^2+^ levels. This activation of PKC and calcium-dependent kinases may lead to phosphorylation of NMDAR subunits, thereby enhancing glutamatergic currents (Garraway and Huie, 2016). In light of these findings, we hypothesize that Mg^2+^ ions may help reduce BDNF-induced excitotoxicity. Given the interaction between MOR and NMDAR in the PAG (Rodríguez-Muñoz et al., 2012) and the expression of BDNF and TrkB receptors (Yin et al., 2014), we can examine the effect of Mg^2+^ ions on morphine analgesia and BDNF levels. Here we tested the hypothesis that magnesium enhances the antinociceptive effects of morphine in streptozotocin-induced diabetic neuropathy and is associated with changes in glutamatergic signaling and neuronal excitability within vlPAG.

## 2 Materials and methods

### 2.1 Animals

All experiments were performed on male Wistar rats (230–290 g). The animals were housed in rooms maintained at 55% ± 10% humidity, 21 °C ± 2 °C, under a 12-h light-dark cycle, with *ad libitum* access to water and food. Diabetic rats were fasted for 16 h prior to STZ injection. Only male Wistar rats were used to avoid variability related to the estrous cycle. Experimental groups consisted of six rats each (n = 6). Group sizes were selected in accordance with the NC3Rs principles. All procedures conformed to Directive 2010/63/EU and were approved by the II Ethical Committee for Experiments on Small Laboratory Animals, Medical University of Warsaw (permit WAW2/110/2021). Animals were randomly assigned to groups.

### 2.2 Experimental protocol

**FIGURE 1:**
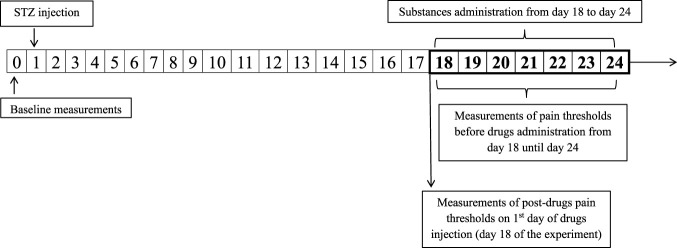
Timeline of the experimental procedure.

Rats were randomly assigned to the following groups: Control/Veh (healthy, saline, “Control”), STZ/Veh (“STZ”), STZ + Mg (magnesium, “STZ + Mg”), STZ + MRF (morphine, “STZ + MRF”), and STZ + Mg + MRF (combination, “STZ + Mg + MRF”). Diabetes was induced by a single intramuscular injection of STZ (40 mg/kg). The development of diabetes was confirmed by measuring blood glucose levels from the tail vein 48 h after STZ injection. Only animals with blood glucose levels ≥ 300 mg/dL were included in further experiments. Moreover, body weight was measured at regular time intervals. STZ-induced diabetic animals demonstrated a progressive reduction in body weight compared to non-diabetic rats. The reduction in body weight did not exceed 15% of baseline values. Magnesium sulfate (40 mg/kg, i. p.) and morphine sulfate (5 mg/kg, i. p.), both dissolved in saline, were administered once daily for seven consecutive days to the designated groups (experimental days 18–24). STZ was administered only on day 1. On the final experimental day, animals were euthanized by decapitation. The brains were rapidly removed, and the PAG was isolated for subsequent analyses. Unless stated otherwise, tissue for *ex vivo* recording was collected after the final injection. Brain slices were prepared 2 h after the final drug administration. The experimental timeline is shown in Figure 1.

### 2.3 Behavior testing

Thermal nociception was assessed using a Plantar Test apparatus (Ugo Basile 37370, Comerio, Italy). Rats were placed in clear boxes on a glass platform, and radiant heat was applied to the left hind paw through the glass. The time between application of the thermal stimulus and paw withdrawal (latency) was automatically recorded and expressed in seconds. Two trials were performed at each time point. Baseline latencies were obtained prior to STZ administration. On the first treatment day (experimental day 18), latencies were measured at 0, 15, 30, 45, 60, 90, and 120 min after injection to assess acute effects. Thereafter, latencies were recorded once daily before dosing for seven consecutive days (days 18–24) to evaluate treatment-dependent changes over time. To prevent tissue injury, the heat stimulus was automatically terminated at the 20 s cut-off latency.

### 2.4 Slice preparation and patch-clamp recordings

Brains were rapidly extracted and placed in ice-cold (0 °C–4 °C), oxygenated protective artificial extracellular solution containing the following (in mM): 125 NaCl, 25 NaHCO_3_, 3 KCl, 1.25 NaH_2_PO_4_, 0.5 CaCl_2_, 6 MgCl_2_, and 25 glucose (bubbled with 95% O_2_/5% CO_2_). Coronal midbrain slices (300 μm thick) containing the ventrolateral PAG were prepared using a vibratome. Electrophysiological recordings were obtained from neurons located in both the left and right ventrolateral PAG. Slices were transferred to a holding solution of artificial cerebrospinal fluid (ACSF) containing (in mM): 125 NaCl, 25 NaHCO_3_, 3 KCl, 1.25 NaH_2_PO_4_, 1 CaCl_2_, 1 MgCl_2_, and 25 glucose, bubbled with 95% O_2_ and 5% CO_2_, adjusted to 320–330 mOsm/kg H_2_O, and incubated at room temperature for at least 60 min.

Slices were transferred to a bath chamber (RC-24E, Warner Instruments, LLC, Hamden, CT, USA) on the stage of an upright microscope (BX51WI, Olympus Corporation, Tokyo, Japan). During recording, slices were perfused with warm (34 °C) extracellular solution containing the following (in mM): 125 NaCl, 25 NaHCO_3_, 3 KCl, 1.25 NaH_2_PO_4_, 1 CaCl_2,_ 1 MgCl_2_, and 25 glucose (bubbled with 95% O_2_/5% CO_2_). Neurons were observed via differential interference contrast microscopy using a ×40 water-immersion objective, a camera (C7500-51), and a camera controller (C2741-62; Hamamatsu Photonics K.K., Hamamatsu City, Japan).

Recording pipettes were pulled from borosilicate glass with a filament (O.D.: 1.5 mm, I.D.: 0.86 mm; Sutter Instrument, Novato, USA) using a P-1000 puller (Sutter Instrument, Novato, USA) and had an open-tip resistance of 4–5 MΩ. For current-clamp recordings of neuronal excitability, pipettes were filled with a K-gluconate–based internal solution (in mM): 130 K-gluconate, 10 KCl, 0.5 ethylene glycol-bis(β-aminoethyl ether)-N,N,N′,N′-tetraacetic acid (EGTA), 10 HEPES, 4 Mg-ATP, 0.4 Na-GTP, adjusted to pH 7.3 with KOH and to 285–295 mOsm/kg H_2_O. For voltage-clamp recordings, a Cs-methanesulfonate–based internal solution was used containing (in mM): 120 CsMeSO_3_, 5 CsCl, 0.5 EGTA, 10 HEPES, 4 Mg-ATP, 0.4 Na-GTP, adjusted to pH 7.3 with CsOH and 285–295 mOsm/kg H_2_O. Liquid-junction potentials were zeroed with the pipette immersed in the bath.

After formation of a giga-seal and membrane rupture by gentle suction, membrane potentials or membrane currents were recorded as appropriate. Signals were low-pass filtered at 1–3 kHz and digitized at 10 kHz. Only neurons with resting membrane potentials between −60 and −79 mV and stable series resistance (<20 MΩ; voltage-clamp compensation 60%–80%) were included. Cells with baseline drift > 10 pA between analysis windows were excluded.

For tonic AMPA recordings, ACSF contained TTX (1 µM) and a GABA_A_ receptor antagonist (picrotoxin, 100 µM), and neurons were held at V_hold_ = - 70 mV. Tonic AMPA current was quantified as the NBQX-sensitive shift in holding current: after a stable baseline, we bath-applied NBQX (a selective AMPA receptor antagonist, 10 µM) and computed ΔI_NBQX_ as the difference between the mean holding current before and after reaching the NBQX plateau. Because AMPA current at −70 mV is inward, NBQX produced an outward shift in the holding current; ΔI_NBQX_ is reported in pA as a positive value. Per-cell values were averaged per animal and used as the unit of analysis.

For NMDA-evoked currents, recordings were performed in ACSF containing NBQX (10 µM) and picrotoxin (100 µM) to isolate NMDA receptors. Neurons were held at −70 mV, and during the test epoch we applied NMDA (2 µM) with glycine (0.05 µM) while delivering a 1-s depolarizing step to −40 mV to partially relieve the Mg^2+^ block. The NMDA-evoked current amplitude was measured at the end of the depolarizing step after reaching a steady level. Specificity was verified by DL-2-Amino-5-phosphonopentanoic acid (DL-AP5, 50 µM), which abolished the response. For current-clamp recordings of neuronal excitability, TTX was omitted from the ACSF to allow action potential generation.

### 2.5 Pharmacological identification of µ-opioid receptor (MOR)-positive vlPAG neurons

To functionally verify µ-opioid receptor (MOR) expression, a subset of vlPAG neurons was tested using the MOR agonist DAMGO ([D-Ala2, NMe-Phe4, Gly-ol5]-enkephalin, 1 µM). In current-clamp recordings, DAMGO typically induced a hyperpolarization of the membrane potential and suppressed step-evoked firing (+200 pA, 1 s). In voltage-clamp recordings at −70 mV, DAMGO elicited an outward holding current consistent with activation of G protein-coupled inwardly rectifying potassium (GIRK) conductance. Specificity was confirmed by naloxone (1 µM), which reversed DAMGO effects. Cells displaying an antagonist-sensitive response were classified as MOR-positive and only these neurons were included in patch-clamp analyses. Neurons that did not display a detectable MOR-mediated response, as well as recordings with unstable baseline parameters or loss of recording quality during the experiment, were excluded from analysis.

### 2.6 ELISA for BDNF concentration

The concentration of BDNF in PAG tissue was determined using a commercial ELISA kit (ab213899, Abcam, UK) according to the manufacturer’s instructions. Samples were processed and assayed following the kit protocol.

### 2.7 Statistical analysis

Data analysis was performed using GraphPad Prism 8 (GraphPad Software, Inc., La Jolla, CA, USA). Results are presented as mean ± SEM. Normality of data distribution was assessed using the Shapiro-Wilk test. Homogeneity of variances was evaluated using the Brown-Forsythe test where appropriate. Depending on the experimental design and fulfillment of statistical assumptions, data were analyzed using one-way or two-way ANOVA models, followed by appropriate multiple-comparison *post hoc* tests. In cases where the assumption of equal variances was violated, Welch’s ANOVA and variance-robust *post hoc* procedures were applied. For repeated-measures analyses, Geisser-Greenhouse correction was used when appropriate. Effect sizes were calculated and reported as partial eta squared (η^2^p) for ANOVA models and Cohen’s d for selected pairwise comparisons. For electrophysiological analyses, recordings obtained from multiple neurons from the same animal were averaged, and the resulting mean value for each animal was used for statistical analysis. Thus, the animal, rather than the individual neuron, was considered the experimental unit. Statistical significance was accepted at p < 0.05.

## 3 Results

### 3.1 Influence of Mg^2+^ on the antinociceptive effect of morphine in diabetic rats after thermal stimulation

**FIGURE 2:**
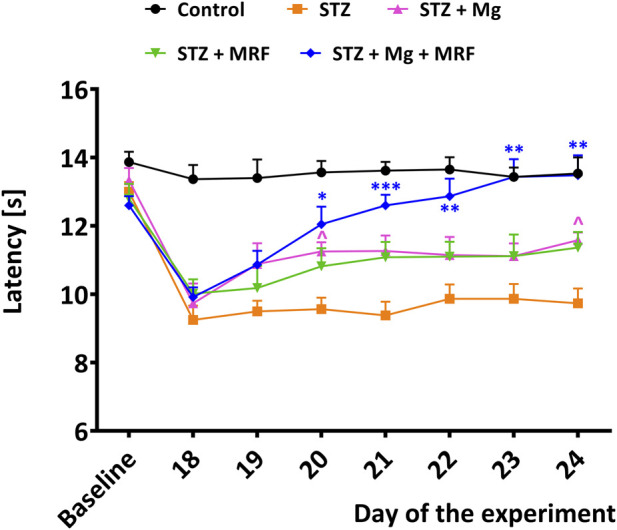
Effect of magnesium on the antinociceptive activity of morphine in neuropathic rats. Pain thresholds, expressed in seconds, were measured before streptozotocin administration (baseline threshold) and then before injection of the tested compounds for 7 consecutive days (corresponding to days 18–24 of the experiment). Data were analyzed using two-way repeated-measures ANOVA followed by Tukey’s multiple comparisons test. Significant differences are indicated as follows: ^***^ p < 0.001; ^**^ p < 0.01; ^*^ p < 0.05 STZ+Mg+MRF vs. STZ and ^ p < 0.05 STZ+Mg vs. STZ. Values are presented as mean ± SEM; n = 6 rats for each group.

In our experiment, we observed a gradual development of thermal hyperalgesia in STZ-injected rodents within 14 days after diabetes induction. A statistically significant increase in magnesium-induced analgesia was recorded on the third (STZ + Mg: 11.3 ± 0.3 s, p < 0.05 vs. STZ) and seventh (STZ + Mg: 11.6 ± 0.3 s, p < 0.05 vs. STZ) day of application (corresponding to days 20 and 24 of the experiment). Importantly, thermal hyperalgesia was significantly reduced in animals treated with the combination of magnesium and morphine starting on day 20 and continuing until the end of the experiment (Figure 2). Two-way repeated-measures ANOVA revealed significant effects of treatment [F (4, 25) = 21.11, p < 0.0001, η^2^p = 0.73], time [F (4.73, 118.2) = 25.20, p < 0.0001, η^2^p = 0.50], and treatment × time interaction [F (18.92, 118.2) = 3.76, p < 0.0001, η^2^p = 0.38]. At the final experimental time point, Cohen’s d values were 2.18 for STZ + Mg vs. STZ and 2.98 for STZ + Mg + MRF vs. STZ.

**FIGURE 3:**
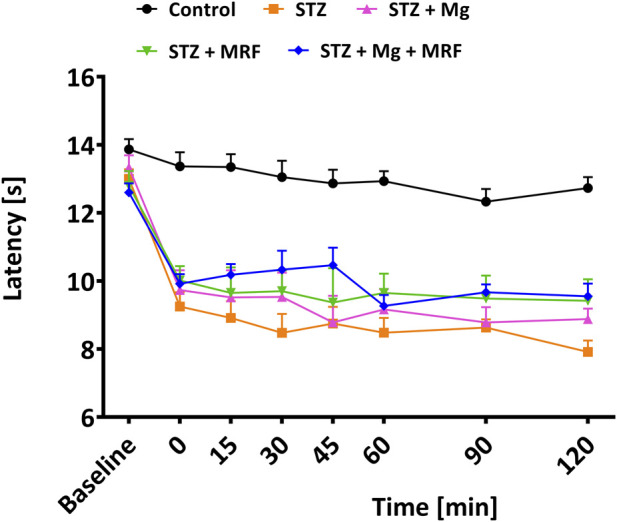
Influence of magnesium, morphine and their combination on the thermal thresholds in tested rats. Nociceptive thresholds, expressed in seconds, were measured before induction of neuropathy (baseline threshold) and then before (0) and after administration of the tested substances for 120 min. Data were analyzed using two-way repeated-measures ANOVA followed by Tukey’s multiple comparisons test. No significant differences were detected between treated and untreated STZ groups at any examined time point. Values are presented as mean ± SEM; n = 6 rats for each group.

Additionally, no changes in STZ-induced hyperalgesia were observed over a 2-h period following the administration of magnesium, morphine, or their combination on the first day of application (day 18 of the experiment) (Figure 3). Two-way repeated-measures ANOVA revealed significant effects of treatment (F (4, 25) = 15.96, p < 0.0001, η^2^p = 0.78), time [F (3.84, 95.90) = 51.23, p < 0.0001, η^2^p = 0.67], and treatment × time interaction [F (28, 175) = 2.49, p = 0.0002, η^2^p = 0.29]. However, *post hoc* analysis revealed no significant differences between STZ-treated animals receiving magnesium, morphine, or their combination and untreated STZ animals at any time point.

Behaviorally, the combination of magnesium and morphine did not produce a significant acute antihyperalgesic effect after the first dose but yielded sustained antinociception across days 20–24. To identify cellular substrates of this efficacy, we performed *ex vivo* whole-cell recordings from vlPAG neurons after the last treatment.

### 3.2 Changes in the action potential firing in diabetic rats treated with magnesium and morphine

**FIGURE 4:**
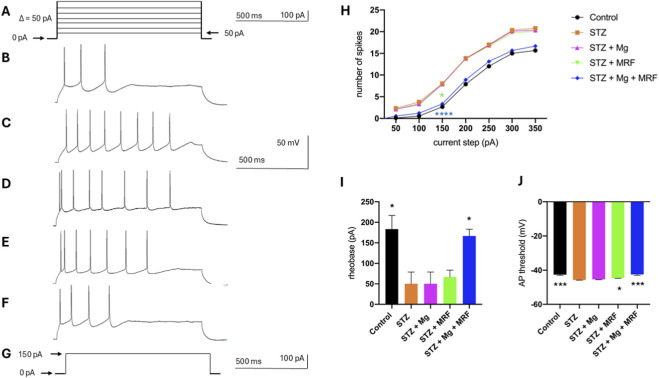
Intrinsic excitability of vlPAG neurons. **(A)** Recording protocol. Schematic of the step protocol used for I-F measurements: 1-s depolarizing current steps from +50 pA to +350 pA in 50-pA increments delivered from the zero-current level. Representative traces evoked by a +150 pA current injection (1 s) **(B)** Control, **(C)** STZ, **(D)** STZ + Mg, **(E)** STZ + MRF, **(F)** STZ + Mg + MRF, **(G)** stimulation protocol used to evoke the traces shown in panels B-F. **(H)** I-F curves obtained from + 50 pA to +350 pA. **(I)** Rheobase (pA). **(J)** Action potential threshold (mV). Statistical analysis of I-F curves **(H)** was performed using two-way repeated-measures ANOVA followed by Dunnett’s multiple comparisons test. Significant differences at the representative current injection of +150 pA are indicated as follows: ^****^p < 0.0001, ^*^p < 0.05 vs. STZ. Statistical analysis of rheobase **(I)** and action potential threshold **(J)** was performed using one-way ANOVA followed by Tukey’s multiple comparisons test. ^***^p < 0.001, ^*^p < 0.05 vs. STZ. Values are presented as mean ± SEM; n = 6 animals per group. Scale bars: 500 ms, 100 pA **(A,G)**; 500 ms, 50 mV **(B–F)**.

Whole-cell current-clamp recordings were obtained using 1-s depolarizing steps from +50 pA to +350 pA (50-pA increments; Figure 4A). Control neurons exhibited graded, intensity-dependent firing. By contrast, STZ neurons showed a significant leftward shift of the I-F relationship relative to Control, indicative of increased intrinsic excitability (STZ vs. Control, p < 0.0001; Figures 4B,C,H). Magnesium alone did not significantly alter the enhanced firing observed in STZ neurons (STZ vs. STZ + Mg, p > 0.05; Figures 4D,H), whereas morphine alone significantly reduced neuronal firing (STZ vs. STZ + MRF, p < 0.05; Figures 4E,H). The combined treatment produced the strongest normalization toward Control (STZ vs. STZ + Mg + MRF, p < 0.0001; Figures 4F,H), with firing rates approaching control values at intermediate and higher current injections. For illustration, at +150 pA the mean spike counts (± SEM) were: Control (2.7 ± 0.1, n = 6), STZ (8.0 ± 0.3, n = 6), STZ + Mg (7.9 ± 0.2, n = 6), STZ + MRF (7.1 ± 0.1, n = 6), STZ + Mg + MRF (3.3 ± 0.1, n = 6) spikes. Two-way repeated-measures ANOVA revealed significant effects of treatment [F (4, 25) = 1964, p < 0.0001], injected current [F (2.17, 54.25) = 11,685, p < 0.0001], and treatment × current interaction [F (8.68, 54.25) = 22.84, p < 0.0001].

Consistent with the left-shifted I-F relationship, STZ neurons exhibited a significantly lower rheobase (50 ± 18.3 pA, n = 6) than Control neurons (183.3 ± 21.1 pA, n = 6; p < 0.05, Figure 4I), indicating facilitated spike initiation. STZ + Mg (50 ± 18.3 pA, n = 6) and STZ + MRF (66.7 ± 10.5 pA, n = 6) did not differ significantly from STZ (p > 0.05 for both). By contrast, the combination of magnesium and morphine (STZ + Mg + MRF) significantly increased rheobase (166.7 ± 10.5 pA, n = 6) relative to STZ (STZ vs. STZ + Mg + MRF, p < 0.05), partially normalizing this parameter toward Control. One-way ANOVA revealed a significant effect of treatment on rheobase (F (4, 25) = 16.48, p < 0.0001, η^2^p = 0.73). Effect size analysis yielded Cohen’s d values of 2.76 for STZ vs. Control and 3.21 for STZ vs. STZ + Mg + MRF.

In line with the left-shifted I-F curves, STZ neurons displayed a more negative action potential threshold than Control neurons (−45.7 ± 0.2 mV, n = 6 vs. −42.6 ± 0.3 mV, n = 6; p < 0.001; Figure 4J). STZ + Mg (−45.3 ± 0.4 mV, n = 6) did not differ from STZ (p > 0.05). STZ + MRF partially normalized the action potential threshold (−44.3 ± 0.2 mV, n = 6; p < 0.05), whereas STZ + Mg + MRF produced the strongest shift toward Control (−42.5 ± 0.3 mV, n = 6; p < 0.001). One-way ANOVA revealed a significant effect of treatment on action potential threshold (F (4, 25) = 46.09, p < 0.0001, η^2^p = 0.88).

### 3.3 NBQX-sensitive tonic current in vlPAG

**FIGURE 5:**
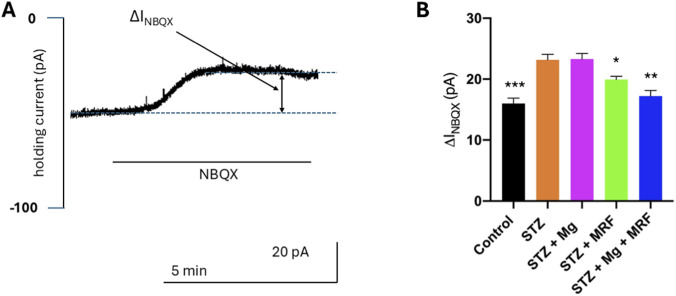
NBQX-sensitive tonic current in vlPAG. **(A)** Representative voltage-clamp trace at V_hold_ = −70 mV. Bath application of NBQX (10 µM) produces an outward shift in the holding current, revealing the NBQX-sensitive tonic AMPA component (ΔI_NBQX_). **(B)** Summary of ΔI_NBQX_ (pA). Statistical analysis was performed using Welch’s ANOVA followed by Dunnett’s multiple comparisons test. ^***^ p < 0.001, ^**^ p < 0.01, ^*^ p < 0.05 vs. STZ. Values are presented as mean ± SEM; n = 6 animals per group. Scale bar: 5 min, 20 pA **(A)**.

At a holding potential of −70 mV, application of NBQX (10 µM) produced an outward shift in the holding current, revealing a NBQX-sensitive tonic AMPA component (ΔI_NBQX_) (Figure 5A). ΔI_NBQX_ was significantly larger in STZ rats (23.2 ± 0.9 pA, n = 6) than in Control animals (16.0 ± 0.9 pA, n = 6), indicating an increase in the NBQX-sensitive tonic current in diabetic rats (STZ vs. Control, p < 0.0001, Figure 5B). Magnesium alone (STZ + Mg, 23.3 ± 1.0 pA, n = 6) did not significantly reduce ΔI_NBQX_ relative to STZ (p > 0.05), whereas morphine alone (STZ + MRF, 19.9 ± 0.5 pA, n = 6) produced a partial reduction (STZ vs. STZ + MRF, p < 0.05). The combined treatment (STZ + Mg + MRF) produced the most pronounced reduction in ΔI_NBQX_ (17.2 ± 0.9 pA, n = 6) relative to STZ (p < 0.001), with values approaching those observed in Control animals (Figure 5B). Welch’s ANOVA revealed a significant effect of treatment on ΔI_NBQX_ (W (4, 12.26) = 11.73, p = 0.0004). Effect size analysis yielded Cohen’s d values of 3.25 for STZ vs. Control, 2.06 for STZ vs. STZ + MRF, and 2.70 for STZ vs. STZ + Mg + MRF.

### 3.4 NMDA-evoked currents in vlPAG

**FIGURE 6:**
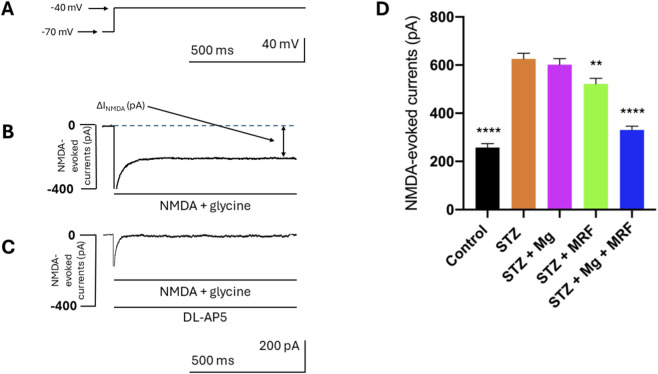
NMDA-evoked currents in vlPAG neurons. **(A)** Schematic illustration of the recording protocol. Neurons were voltage-clamped at −70 mV, and NMDA (2 µM) with glycine (0.05 µM) was bath-applied during a 1-s depolarizing step to −40 mV. Representative voltage-clamp traces showing baseline conditions, NMDA-evoked currents during the depolarizing step **(B)**, and blockade of the response by DL-AP5 (50 µM) **(C)**. **(D)** Summary of NMDA-evoked current amplitudes (pA). Statistical analysis was performed using one-way ANOVA followed by Tukey’s multiple comparisons test. ^****^ p < 0.0001, ^**^ p < 0.01 vs. STZ. Values are presented as mean ± SEM; n = 6 animals per group. Scale bars: 500 ms, 40 mV **(A)**; 500 ms, 200 pA **(B,C)**.

To isolate NMDAR responses, neurons were voltage-clamped in ACSF containing NBQX (10 µM) and picrotoxin (100 µM). NMDA (2 µM) with glycine (0.05 µM) was bath-applied during a 1-s depolarizing step to −40 mV (Figures 6A,B); amplitudes were quantified at the end of the step and were abolished by DL-AP5 (50 µM), confirming the specificity of the recorded currents (Figure 6C). STZ rats displayed significantly larger NMDA-evoked currents (625.3 ± 23.9 pA, n = 6) than Control animals (257.7 ± 16.2 pA, n = 6; STZ vs. Control, p < 0.0001, Figure 6D). Magnesium alone (STZ + Mg, 601.3 ± 25.5 pA, n = 6) reduced the group mean relative to STZ but not significantly (p > 0.05). Morphine alone (STZ + MRF, 521.5 ± 23.8 pA, n = 6) significantly reduced NMDA-evoked current amplitudes compared with the STZ group (STZ vs. STZ + MRF, p < 0.01). The combination (STZ + Mg + MRF, 330.5 ± 15.7 pA, n = 6) resulted in the largest reduction relative to STZ (p < 0.0001), with NMDA-evoked current amplitudes approaching those observed in the Control group (Figure 6D). One-way ANOVA revealed a significant effect of treatment on NMDA-evoked current amplitudes (F (4, 25) = 59.16, p < 0.0001, η^2^p = 0.90).

### 3.5 Changes in the level of BDNF in diabetic rats treated with magnesium and morphine

**FIGURE 7:**
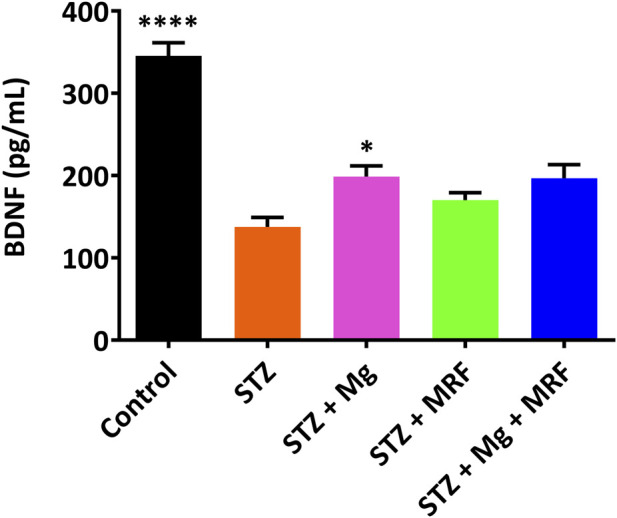
Changes in BDNF concentration in periaqueductal grey. Statistical analysis was performed using Welch’s ANOVA followed by Dunnett’s T3 multiple comparisons test. ^****^ p < 0.0001, ^*^ p < 0.05 vs. STZ. Values are presented as mean ± SEM; n = 6 animals per group.

In our study, a statistically significant decrease in BDNF levels was observed in animals with STZ-induced diabetes compared to the control group (STZ: 137.6 ± 11.6 pg/mL vs. Control: 345.5 ± 16.0 pg/mL, p < 0.0001). Morphine administered alone for seven consecutive days did not significantly alter BDNF concentrations compared to untreated diabetic rats. In contrast, 7-day administration of magnesium significantly increased BDNF levels relative to STZ-treated animals (STZ + Mg: 198.8 ± 13.1 pg/mL, p < 0.05 vs. STZ) (Figure 7). Welch’s ANOVA revealed a significant effect of treatment on BDNF levels (W (4, 12.31) = 25.90, p < 0.0001).

## 4 Discussion

Chronic pain management remains insufficient for many patients, highlighting the need for new therapeutic approaches.

The results of this study indicate that in rats with STZ-induced diabetes, co-administration of magnesium and morphine was associated with enhanced antihyperalgesic effect, reaching values similar to those observed in control rats in the final days of the experiment. This effect was accompanied by convergent cellular changes in vlPAG, including reduced neuronal excitability and attenuation of glutamatergic current components. This behavioral improvement was accompanied by attenuation of neuronal hyperexcitability in the PAG, a key brainstem region involved in descending pain modulation. Whereas morphine alone reduced action potential frequency and inward current amplitudes, the combined treatment (Mg + MRF) affected a broader range of electrophysiological measures, including the I–F relationship, rheobase, action potential threshold, NBQX-sensitive tonic current, and NMDA-evoked currents.

Our studies revealed a gradual decrease in the thermal pain threshold in diabetic rats, which indicates the development of hyperalgesia. Furthermore, diabetes induced by STZ was associated with increased excitability of PAG neurons. This conclusion is supported by the observed enhancement of action potential generation and increased inward and NMDA-evoked currents. These electrophysiological changes may contribute to behavioral hyperalgesia and are consistent with previous reports of enhanced excitatory transmission and reduced inhibitory tone in both spinal and supraspinal regions under diabetic conditions (Afrazi and Esmaeili-Mahani, 2014; Morgado et al., 2008). Furthermore, increased NMDAR activity has been implicated in the pathophysiology of diabetic neuropathic pain (Calcutt, 2002), highlighting the role of altered glutamatergic signaling and impaired inhibition. Although the increase in NBQX-sensitive holding current is consistent with altered tonic glutamatergic signaling, extracellular glutamate levels, glutamate transporter activity, and spontaneous synaptic transmission were not assessed in the present study. Therefore, the mechanisms underlying these changes remain to be determined. Notably, our experiments revealed a significant decrease in BDNF concentration in the PAG on day 25 following diabetes induction. Similarly, Nitta et al. reported reduced BDNF levels in the cerebral cortex and hippocampus of rats 4 weeks after STZ injection (Nitta et al., 2002). In contrast, some studies have observed elevated neurotrophin concentrations in the spinal cord and skeletal muscle of diabetic rats (Fernyhough et al., 1995; Ismail et al., 2021). In the study by Miao et al., the highest BDNF levels in the DRG and spinal cord were observed on day 21 after STZ injection, with BDNF levels decreasing by day 28 (Miao et al., 2021). Based on our findings and existing literature, we suggest that these discrepancies in reported BDNF level changes across studies may result from differences in diabetes duration, analysis time points, and the specific structures studied. Measuring BDNF levels at various time points could provide more precise insights into changes in neurotrophin synthesis.

The present data confirm that morphine administration significantly reduced neuronal excitability in diabetic rats, consistent with previous studies showing MOR-mediated suppression of neuronal activity in various brain regions (North et al., 1987; Vaughan et al., 1997). This suppression is believed to result from multiple mechanisms, including inhibition of voltage-gated N-type calcium channels, activation of G-protein-coupled inwardly rectifying potassium channels, and a reduction in presynaptic glutamate release (Williams et al., 2001). Nevertheless, the behavioral efficacy of morphine in STZ rats was limited. In our behavioral experiments, morphine did not produce an acute antihyperalgesic effect after the first administration, and its effects during repeated treatment remained modest compared with the combined Mg + MRF treatment. A reduced responsiveness to morphine in diabetic rats has also been observed in our previous studies using mechanical nociceptive stimuli (Kulik et al., 2021). Begon et al. showed a slight antinociceptive effect in animals with sciatic nerve ligation treated with morphine at a dose of 0.3 mg/kg, but intravenous injection of morphine at a dose of 1 mg/kg did not alter the nociceptive threshold in diabetic rats (Begon et al., 2002). Moreover, our present research demonstrated that morphine treatment had no significant effect on BDNF concentration in the PAG of diabetic rats. Other studies report conflicting findings. Brazile et al. observed increased BDNF levels in mice treated with morphine for 30 days (Brazile et al., 2024), whereas Kosciuczuk et al. reported decreased BDNF concentrations in patients with chronic low back pain undergoing long-term opioid therapy (median duration: 26 months) (Kosciuczuk et al., 2022). These findings suggest that BDNF levels may depend on the duration of opioid exposure.

A key observation in this study is that co-administration of morphine and magnesium sulfate produced a more substantial reduction in NMDA-evoked currents in diabetic animals compared to either agent alone. Magnesium, a voltage-dependent NMDAR antagonist (Nowak et al., 1984), did not significantly alter baseline excitability. In our voltage-clamp protocol, brief depolarization to −40 mV partially relieves the Mg^2+^ pore block; Mg given alone reduced group means non-significantly, whereas adding MOR activation (morphine) yielded the largest attenuation. However, under depolarizing conditions, it was associated with a significant reduction in NMDA-evoked currents. These findings align with previous studies indicating that the efficacy of magnesium is dependent on membrane potential (Mayer et al., 1984). The limitation of the present NMDA-evoked current is that exogenous NMDA/glycine application during depolarization does not permit discrimination between changes in receptor expression, voltage-dependent properties, magnesium sensitivity, or downstream intracellular signaling mechanisms. Therefore, the observed changes in NMDA-evoked currents following treatment may reflect several complementary mechanisms rather than a single specific effect on NMDAR function. Our behavioral data also showed a modest antihyperalgesic effect of magnesium sulfate, observed on the third and seventh days of ion administration in rats with established hyperglycemia (STZ + Mg vs. STZ; p < 0.05). The literature provides numerous studies indicating a dose-dependent analgesic effect of Mg^2+^ in alleviating various types of neuropathic pain (Begon et al., 2002; Rondon et al., 2010). Furthermore, the effect was similar to that of synthetic NMDAR antagonists (Begon et al., 2000; Bujalska-Zadrożny and Duda, 2014). In particular, Rondon et al. demonstrated that oral administration of magnesium in drinking water for 3 weeks significantly alleviated mechanical hyperalgesia in animals (Rondon et al., 2010). Consistent with an interaction between BDNF signaling and glutamatergic gain, our present experiment also showed a significant increase in BDNF concentration following magnesium administration. BDNF has been shown to promote phosphorylation of tyrosine residues on the NR1 and NR2 subunits of NMDARs, thereby enhancing receptor activity and contributing to the maintenance of neuropathic pain (di Luca et al., 2001; Levine et al., 1998). Since Mg^2+^ ions block NMDARs, they may inhibit the pronociceptive effects of BDNF. As mentioned above, our pain sensitivity assessments support this suggestion, demonstrating the protective role of magnesium in STZ-induced hyperalgesia.

Notably, diabetic rats treated with both morphine and magnesium sulfate exhibited a greater reduction in NMDA-evoked currents than animals treated with either compound. The increased antihyperalgesic effect of the combined treatment was also observed from day 20 until the last day of the experiment. Data from the literature confirm the efficacy of combined opioid and magnesium administration, as well as synthetic NMDAR antagonists, in chronic neuropathic pain (Begon et al., 2002; Martinez et al., 2022; Ulugol et al., 2002).

The present findings suggest that, although magnesium sulfate has limited analgesic effects when administered alone, its analgesic effect becomes noticeable after it is combined with morphine. This effect may be associated with changes in neuronal excitability and glutamatergic signaling, including attenuation of NMDA-evoked currents, although the precise cellular mechanisms remain to be determined. The enhanced effect observed in our study is consistent with previous reports demonstrating magnesium’s ability to potentiate opioid analgesia in various pain models (Do, 2013; Kulik et al., 2021).

Furthermore, these findings support further investigation of approaches targeting both opioid and glutamatergic mechanisms in diabetic neuropathy. Modulation of neuronal excitability and excitatory neurotransmission may represent a promising area for future preclinical and translational research.

In conclusion, the present study demonstrates that co-administration of magnesium sulfate with morphine increased the antihyperalgesic effect in STZ-induced diabetic rats, reaching values similar to those observed in control animals. At the cellular level, the combination was associated with the greatest reduction in NMDA-evoked currents and with intrinsic excitability parameters approaching those observed in control animals, suggesting that targeting both opioid and glutamatergic systems may represent a promising direction for future research aimed at improving pain management in diabetic neuropathy.

## Data Availability

The original contributions presented in the study are included in the article/supplementary material, further inquiries can be directed to the corresponding author.
